# Paralogue annotation identifies novel pathogenic variants in patients with Brugada syndrome and catecholaminergic polymorphic ventricular tachycardia

**DOI:** 10.1136/jmedgenet-2013-101917

**Published:** 2013-10-17

**Authors:** Roddy Walsh, Nicholas S Peters, Stuart A Cook, James S Ware

**Affiliations:** 1 NIHR Royal Brompton Cardiovascular Biomedical Research Unit, Royal Brompton and Harefield NHS Trust, London, UK; 2 National Heart and Lung Institute, Imperial College, London, UK; 3 Cardiovascular & Metabolic Disorders, Duke National University of Singapore, Singapore, Singapore; 4 National Heart Centre Singapore, Singapore, Singapore

**Keywords:** Genetics, Cardiovascular Medicine, Clinical Genetics, Congenital Heart Disease

## Abstract

**Background:**

Distinguishing genetic variants that cause disease from variants that are rare but benign is one of the principal challenges in contemporary clinical genetics, particularly as variants are identified at a pace exceeding the capacity of researchers to characterise them functionally.

**Methods:**

We previously developed a novel method, called paralogue annotation, which accurately and specifically identifies disease-causing missense variants by transferring disease-causing annotations across families of related proteins. Here we refine our approach, and apply it to novel variants found in 2266 patients across two large cohorts with inherited sudden death syndromes, namely catecholaminergic polymorphic ventricular tachycardia (CPVT) or Brugada syndrome (BrS).

**Results:**

Over one third of the novel non-synonymous variants found in these studies, which would otherwise be reported in a clinical diagnostics setting as ‘variants of unknown significance’, are categorised by our method as likely disease causing (positive predictive value 98.7%). This identified more than 500 new disease loci for BrS and CPVT.

**Conclusions:**

Our methodology is widely transferable across all human disease genes, with an estimated 150 000 potentially informative annotations in more than 1800 genes. We have developed a web resource that allows researchers and clinicians to annotate variants found in individuals with inherited arrhythmias, comprising a referenced compendium of known missense variants in these genes together with a user-friendly implementation of our approach. This tool will facilitate the interpretation of many novel variants that might otherwise remain unclassified.

## Introduction

Inherited arrhythmias such as long QT syndrome (LQTS), Brugada syndrome (BrS) and catecholaminergic polymorphic ventricular tachycardia (CPVT) are life-threatening diseases, caused predominantly by genetic variation in ion channels. In BrS, loss-of-function mutations in the *SCN5A*-encoded cardiac sodium channel (MIM 601144) have been shown to be responsible for 15–30% of cases^1^ ^2^ with mutations in other minor genes accounting for a proportion of remaining cases. In CPVT, gain-of-function mutations in the cardiac ryanodine receptor encoded by *RYR2* (MIM 604772) are responsible for at least 50% of cases.^3^

With on-going developments in DNA sequencing technology, it is expected that clinical genetic testing will become very widely available. However, it is increasingly appreciated that many healthy individuals carry rare variants in disease-associated genes (3–6% for genes associated with inherited arrhythmia syndromes),^4^ ^5^ and that additional information is required to determine whether a novel variant identified during genetic testing is pathogenic. This is particularly the case for missense variants (single amino acid substitutions) caused by single nucleotide variants.

In order to determine whether a rare variant found in a patient is disease causing, sufficiently powered segregation analysis or functional biochemical characterisation are ideally performed. However, these are often impractical due to cost and time constraints, or a lack of phenotypically characterised family members for segregation studies. Several in silico algorithms, such as SIFT and Polyphen^6^ ^7^ try to predict the effect of novel variants, based on the conservation and physicochemical properties of the variant amino acid, and variants in certain protein regions and domains have a high probability of pathogenicity.^8^ ^9^ However, more evidence is needed to classify variants with sufficient confidence for clinical application.

**Figure 1:**
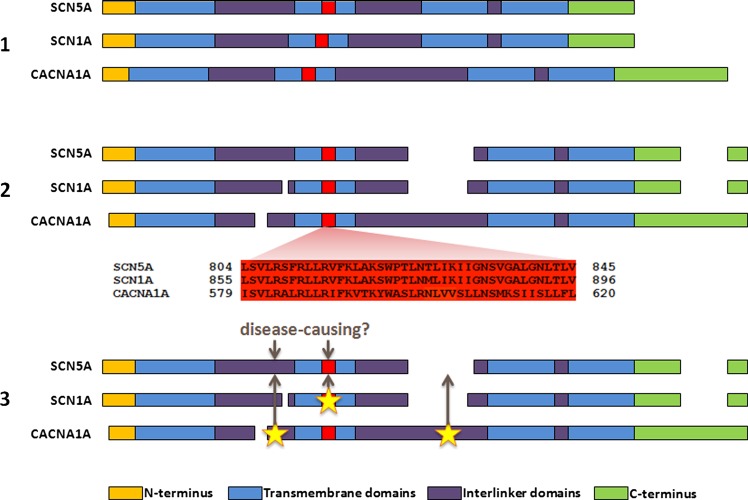
An overview of paralogue annotation. (1) Paralogues (evolutionarily related genes, with homologous sequence and protein domain structures) are identified for a gene of interest. A subset of paralogues for *SCN5A* is shown here for illustration. (2) Protein sequences of paralogues are aligned, identifying functionally equivalent amino acids across the protein family. (3) Disease-causing variants in paralogues are identified from previous literature reports, and their locations are mapped to the gene of interest. Variants at these sites have a high probability of pathogenicity.

We have recently proposed a new method to analyse novel missense variants and assess their likelihood of pathogenicity.^10^ This method, ‘paralogue annotation’, identifies functionally important residues that are intolerant of variation across families of evolutionarily related proteins (paralogues), using clinically ascertained human genotype–phenotype relationships. By aligning the protein sequences of members of these protein families, we can identify amino acids that are functionally equivalent across the different proteins. A variant that is known to be pathogenic in one member of a protein family can then be used to annotate the equivalent amino acid of other members of the family for which no clinical genetic information exists (figure 1). For example, if a missense variant in *RYR1* alters protein function and causes malignant hyperthermia when expressed in skeletal muscle, then we hypothesise that a novel variant affecting the equivalent amino acid in *RYR2*, expressed in cardiac muscle, is likely to be disease causing in a patient with CPVT.

This approach was developed and experimentally validated by application to a large set of known variants in eight LQTS genes, and was found to have a positive predictive value (PPV) of 98.4% in these genes.^10^ Here we present novel refinements to increase accuracy, and apply this approach in two large cohorts of patients with BrS or CPVT to determine whether it provides additional useful information in a clinical diagnostic setting. We also report a web application that allows researchers and clinicians to use paralogue annotation to interrogate novel variants in arrhythmia genes.

## Materials and methods

### Identification of variants in paralogues of *RYR2* and *SCN5A*

Paralogues of *RYR2* and *SCN5A*, that is, evolutionarily related genes with homology in sequence and structure, were identified using the IUPHAR database on receptor nomenclature^11^ and through homology searches (Blastp searches of the *SCN5A* and *RYR2* protein sequences against the human Refseq protein database).^12^ The transcripts and protein isoforms used for these genes were *RYR2*: NM_001035/NP_001026 (Refseq), ENST00000366574/ENSP00000355533 (Ensembl), LRG_402t1/LRG_402p1 (Locus Reference Genomic) and SCN5A: NM_198056/NP_932173 (Refseq), ENST00000333535/ENSP00000328968 (Ensembl), LRG_289t1/LRG_289p1 (Locus Reference Genomic).

*RYR2* has two paralogues, *RYR1* and *RYR3*, which were included in the sequence alignment. Although *RYR3* is not implicated in human disease it was included to improve the alignment quality for the protein family. Variants in *RYR1* cause malignant hyperthermia, central core disease, multi-minicore disease and congenital myopathy. There are 329 *RYR1* missense variants in the Human Gene Mutation Database (HGMD) affecting 284 distinct amino acid residues.

For *SCN5A*, 19 paralogues were included: nine voltage-gated sodium channels, and 10 voltage-gated calcium channels that show strong sequence and structural homology with the sodium channels. Fourteen of these 19 paralogues are associated with Mendelian disease in humans, typically attributable to altered cellular electrophysiology in the tissue where the paralogue is expressed, such as epilepsy, myotonia, pain disorders, night blindness and hemiplegic migraine. *SCN5A* paralogues and their disease associations are shown in table 1.

**Table 1:** *SCN5A* paralogues

Paralogue	HGMD missense mutations	HGMD missense residues	*SCN5A* annotations	Major diseases
*SCN1A*	410	327	393	Epilepsy, Dravet syndrome, Hemiplegic migraine
*SCN2A*	26	26	24	Epilepsy, Neonatal-infantile seizures
*SCN3A*	1	1	1	Epilepsy
*SCN4A*	67	53	67	Hyperkalaemic periodic paralysis, myotonia, paramyotoniacongenita, periodic paralysis
*SCN7A*	0	0	0	
*SCN8A*	1	1	1	Infantile epileptic encephalopathy
*SCN9A*	53	50	45	Congenital indifference to pain, primary erythermalgia, paroxysmal extreme pain disorder, Small fibre neuropathy
*SCN10A*	0	0	0	
*SCN11A*	0	0	0	
*CACNA1A*	63	61	54	Episodic ataxia 2, hemiplegic migraine
*CACNA1B*	0	0	0	
*CACNA1C*	9	9	4	BrS, LQTS
*CACNA1D*	1	1	0	
*CACNA1E*	1	1	0	
*CACNA1F*	28	28	27	Night blindness
*CACNA1G*	2	2	0	Juvenile myoclonic epilepsy
*CACNA1H*	25	25	13	Epilepsy, autism spectrum disorder
*CACNA1I*	0	0	0	
*CACNA1S*	13	8	13	Hypokalaemic periodic paralysis, malignant hyperthermia

Nineteen paralogues of SCN5A were used in this study, including voltage-gated sodium channels (SCN-A) and the homologous voltage-gated calcium channels. For each paralogue, the table shows the total number of distinct missense variants reported in HGMD, the number of distinct amino acid residues affected by these mutations and the number of mutations that were mapped to equivalent residues in SCN5A. Some of the most prominent diseases associated with these paralogue mutations are also highlighted: these are typically diseases attributable to abnormalities of membrane excitability in a range of tissues.

BrS, Brugada syndrome; HGMD, Human Gene Mutation Database; LQTS, long QT syndrome.

For each paralogue gene, variants reported as pathogenic were identified using the HGMD Professional V.2012.3.^13^ Only disease-causing missense mutations, that is, single nucleotide variants causing a single non-terminal amino acid change, were considered.

### Multiple sequence alignment and paralogue annotation

The protein sequences of *RYR2* and *SCN5A* and their respective paralogues were aligned using the T-Coffee/M-Coffee alignment packages.^14^ These packages combine the output of a number of alignment algorithms into a single consensus alignment and provide a consensus score at each point in the alignment (0–9), which is a measure of the reliability of the alignment at each amino acid residue. Using these alignments, each paralogue protein residue with a disease-causing variant was mapped onto the equivalent amino acid residue in *RYR2* and *SCN5A*.

To distinguish aligned amino acids that are truly functionally equivalent from alignment artefacts, an amino acid alignment quality classification was devised. Mappings were classified as high quality if the reference amino acid was conserved between paralogues, and the alignment consensus score was greater than 3. Medium quality mappings required conservation of the reference amino acid or a consensus score greater than 3 or more than one paralogue variant mapping to the same residue. Low quality mappings did not meet these criteria, and were excluded from analyses.

### Sequencing and paralogue annotation in cohorts with CPVT and BrS

Sequencing of two large cohorts of unrelated patients with inherited arrhythmias has previously been reported: all exons of *RYR2* were sequenced in 155 patients with CPVT and 200 healthy controls,^4^ and all exons of *SCN5A* were sequenced in 2111 patients with BrS and 1300 healthy controls.^5^ Paralogue annotation was applied to novel missense variants identified in each cohort. Variants were classified as ‘novel’ if they had not previously been reported at the time of the original report, and have not subsequently been confirmed in an independent study. These would conventionally be reported clinically as ‘variants of unknown significance’, pending segregation and/or functional molecular characterisation.

### Mutation status of *RYR2* and *SCN5A* residues

Previous reports of benign and pathogenic variation in *RYR2* and *SCN5A* were collated. HGMD identified putative disease-causing variants in both genes. Variants were also extracted from dbSNP V.137 as part of the Ensembl 69 release. Population frequency from 1000 Genomes (phase 1) and other datasets in dbSNP were also used—any variant with more than one observation and a minor allele frequency (MAF) of greater than or equal to 0.01 in any population was classified as benign, as this is considered incompatible with the population frequencies of BrS and CPVT. In addition, several large published datasets of *RYR2* and *SCN5A* pathogenic and benign variants were assessed.^15–20^

Residues with one or more missense variants reported as disease causing were classified as pathogenic. Residues with reported benign missense variants or high MAF scores as described above were classified benign. Any residue with conflicting benign and pathogenic reports (for the same variant or different variants affecting the same residue), or dbSNP variants with no population frequency data or MAF of less than 0.01, were classified as uncertain. Residues for which no missense variants were known were classified as unannotated.

### Protein domains of *RYR2* and *SCN5A*

Mutation hotspots in *RYR2* were defined according to Yano *et al*:^9^ residues 77–466 (N-terminal hotspot), 2246–2534 (central hotspot) and 3778–4959 (channel hotspot). The *SCN5A* domains were defined in accordance with the Uniprot entry Q14524 as used by Kapa *et al*:^8^ transmembrane regions are between residues 127–415, 712–939, 1201–1470 and 1524–1772.

## Results

### Paralogue annotation is informative in assessing novel missense variants in clinical cohorts

**Figure 2:**
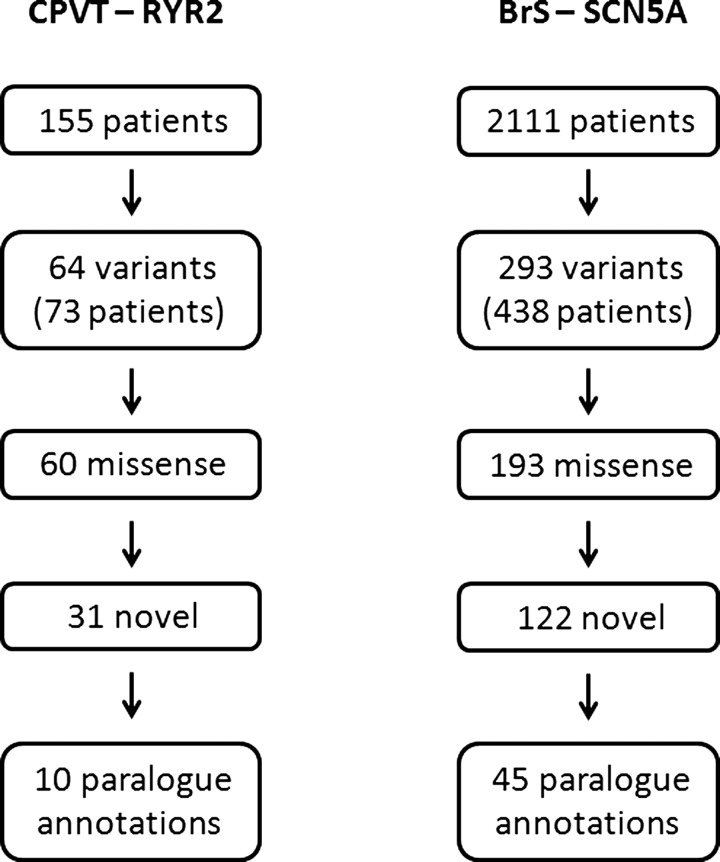
Genetic variation in cohorts with inherited arrhythmia syndromes. *RYR2* or *SCN5A* were sequenced in 2266 patients with an inherited arrhythmia syndrome, as shown. Three hundred and fifty-seven variants were identified in 511 patients, of which 153 were novel missense variants. In the absence of segregation or functional data, these would typically be reported as variants of unknown significance. However, paralogue annotation provided additional information for 65 (36%) variants, that would further inform a clinical genetic report. BrS, Brugada syndrome; CPVT, catecholaminergic polymorphic ventricular tachycardia.

Variants identified in two large cohorts of unrelated patients with inherited arrhythmias were analysed. *RYR2* sequencing in 155 CPVT patients identified 63 distinct variants that were absent in 200 healthy control references. Of these, 59 were missense variants, and 31 were not reported in any other previous or subsequent cases. *SCN5A* sequencing 2111 BrS patients identified 293 distinct *SCN5A* variants that were absent in 1300 healthy controls. Of these, 193 were missense variants, and 122 were not reported in any other previous or subsequent cases. Paralogue annotation was applied to these 31 *RYR2* and 122 *SCN5A* variants, which would otherwise be reported clinically as ‘variants of unknown significance’, to determine whether paralogue mutation annotation is informative and provides further evidence to reclassify some of these variants as ‘disease causing’ (figure 2).

Ten of the 31 novel *RYR2* missense variants (32.3%) affected amino acid residues that were annotated with paralogue mutations, that is, variants in the equivalent residue in *RYR1* have been shown to be disease causing for other human diseases (table 2). Five of these 10 residues have more than one reported *RYR1* pathogenic mutation. Three of these variants occurred in the N-terminal hotspot (residues 77–466), five occurred in the channel region hotspot (residues 3778–4959) and two occurred outside the recognised *RYR2* mutation hotspots—A549 V and H2168Q. In addition, five of the 29 non-novel *RYR2* missense variants identified in the study also had equivalent paralogue mutations, adding further evidence to the likely pathogenicity of these variants (see supplementary table S1, available online only).

**Table 2:** Paralogue annotation of novel variants identified in *RYR2*

*RYR2* variant								
CDS	Protein	Region	Cases (n=155)	Exon	Paralogue	Paralogue variant	Paralogue disease	Consensus
c.527G>A	p.R176Q	N-terminal hotspot	1	8	*RYR1*	R163L	Malignant hyperthermia	9
					*RYR1*	R163C	Central core disease	9
c.994C>T	p.R332W	N-terminal hotspot	1	12	*RYR1*	R316L	Malignant hyperthermia	8
c.1069G>A	p.G357S	N-terminal hotspot	1	13	*RYR1*	G341R	Malignant hyperthermia	9
					*RYR1*	G341R	Malignant hyperthermia	9
c.1646C>T	p.A549V	Outside hotspots	1	17	*RYR1*	A537T	Congenital myopathy	9
c.6504C>G	p.H2168Q	Outside hotspots	2	42	*RYR1*	H2204Q	Multiminicore disease	9
c.7258A>T	p.R2420W	Central hotspot	1	48	*RYR1*	R2454C	Malignant hyperthermia	9
					*RYR1*	R2454H	Malignant hyperthermia	9
c.11989A>G	p.K3997E	Channel hotspot	1	90	*RYR1*	R4041W	Malignant hyperthermia	9
c.14369G>A	p.R4790Q	Channel hotspot	1	100	*RYR1*	R4861C	Central core disease	9
					*RYR1*	R4861H	Central core disease	9
c.14414A>G	p.K4805R	Channel hotspot	1	100	*RYR1*	K4876R	Malignant hyperthermia	9
c.14465G>A	p.R4822H	Channel hotspot	1	101	*RYR1*	R4893W	Central core disease	9
					*RYR1*	R4893Q	Central core disease	9
					*RYR1*	R4893P	Central core disease	9

Ten out of 31 novel missense variants identified in 155 unrelated CPVT patients^10^ were annotated. This provides strong evidence of pathogenicity for these variants. In addition, five of the 29 *RYR2* missense variants previously reported to be pathogenic were annotated (see supplementary table S1, available online only).

*RYR2* coordinates given with respect to transcripts NM_001035/NP_001026 (Refseq), ENST00000366574/ENSP00000355533 (Ensembl), LRG_402t1/LRG_402p1 (Locus Reference Genomic).

CDS, coding DNA sequence; CPVT, catecholaminergic polymorphic ventricular tachycardia.

In *SCN5A*, 45 of the 122 novel missense variants (36.9%) were annotated with paralogue mutations (table 3). Fourteen of these 45 residues have more than one reported paralogue pathogenic mutation. Forty-one of these variants occurred in the transmembrane domains of *SCN5A*, three in the N-terminal region and one in the interlinker domains. In addition, 26 of the 70 non-novel *SCN5A* missense variants identified in the study also had equivalent paralogue mutations, adding further evidence to the likely pathogenicity of these variants (see supplementary table S2, available online only).

**Table 3:** Paralogue annotation of novel variants identified in *SCN5A*

*SCN5A* variant								
CDS	Protein	Region	Cases (n=2111)	Exon	Paralogue	Paralogue variant	Paralogue disease	Consensus
c.278T>C	p.F93S	N-terminus	1	3	*SCN1A*	F90S	Myoclonic epilepsy of infancy	5
c.281T>G	p.I94S	N-terminus	1	3	*SCN1A*	I91T	Myoclonic epilepsy of infancy	5
c.362G>A	p.R121Q	N-terminus	2	3	*SCN1A*	R118S	Myoclonic epilepsy of infancy	9
c.533C>G	p.A178G	TM domain 1	1	5	*SCN1A*	A175V	Dravet syndrome	9
					*SCN1A*	A175T	Myoclonic epilepsy of infancy	9
c.659C>T	p.T220I	TM domain 1	2	6	*SCN1A*	T217K	Myoclonic epilepsy of infancy	9
c.694G>A	p.V232I	TM domain 1	2	6	*CACNA1H*	R212R	Autism spectrum disorder	9
c.1100G>T	p.R367L	TM domain 1	1	9	*SCN1A*	R377Q	Generalised epilepsy with febrile seizures	9
					*SCN1A*	R377L	Dravet syndrome	9
c.1120T>G	p.W374G	TM domain 1	1	9	*SCN1A*	W384R	Dravet syndrome	9
					*SCN1A*	W384X	Myoclonic epilepsy of infancy	9
c.1157G>A	p.G386E	TM domain 1	2	10	*SCN1A*	G396E	Dravet syndrome	8
c.1156G>A	p.G386R	TM domain 1	1	10	*SCN1A*	G396E	Dravet syndrome	8
c.1187T>C	p.V396A	TM domain 1	1	10	*SCN1A*	V406F	Dravet syndrome	9
c.1186G>C	p.V396L	TM domain 1	1	10	*SCN1A*	V406F	Dravet syndrome	9
c.2047T>G	p.C683G	Interdomain Linker I-II	1	14	*SCN9A*	C699Y	Dravet syndrome	3
					*CACNA1H*	R744Q	Childhood absence epilepsy	3
c.2150C>T	p.P717L	TM Domain 2	1	14	*SCN1A*	P768L	Myoclonic epilepsy of infancy	8
c.2553C>A	p.F851L	TM Domain 2	1	16	*SCN1A*	F902C	Myoclonic epilepsy of infancy	9
c.2633G>A	p.R878H	TM Domain 2	5	16	*SCN1A*	R931C	Myoclonic epilepsy of infancy	8
					*SCN9A*	R896Q	Congenital indifference to pain	8
					*SCN1A*	R931H	Epilepsy	8
c.2657A>C	p.H886P	TM Domain 2	1	16	*SCN1A*	H939Q	Myoclonic epilepsy of infancy	9
					*SCN1A*	H939Y	Dravet syndrome	9
					*CACNA1H*	W962C	Autism spectrum disorder	9
c.2677C>T	p.R893C	TM Domain 2	2	16	*SCN1A*	R946S	Generalised epilepsy of infancy	9
					*SCN1A*	R946C	Myoclonic epilepsy of infancy	9
					*SCN1A*	R946H	Myoclonic epilepsy of infancy	9
c.2678G>A	p.R893H	TM Domain 2	3	16	*SCN1A*	R946S	Generalised epilepsy of infancy	9
					*SCN1A*	R946C	Myoclonic epilepsy of infancy	9
					*SCN1A*	R946H	Myoclonic epilepsy of infancy	9
c.2701G>A	p.E901K	TM Domain 2	3	16	*SCN1A*	E954K	Dravet syndrome	9
c.3695G>A	p.R1232Q	TM Domain 3	1	21	*SCN1A*	R1245Q	Myoclonic epilepsy of infancy	7
c.3758A>G	p.E1253G	TM Domain 3	1	21	*SCN1A*	E1266A	Dravet syndrome C	9
c.3813G>C	p.W1271C	TM Domain 3	1	21	*SCN1A*	W1284S	Dravet syndrome	9
c.3968T>G	p.V1323G	TM Domain 3	1	23	*SCN9A*	V1299F	Paroxysmal extreme pain disorder	9
c.4057G>A	p.V1353M	TM Domain 3	2	23	*SCN1A*	V1366I	Generalised epilepsy with febrile seizures	9
c.4079T>G	p.F1360C	TM Domain 3	1	23	*CACNA1A*	F1404C	Episodic ataxia	9
c.4226A>G	p.Y1409C	TM Domain 3	1	23	*SCN1A*	Y1422C	Myoclonic epilepsy of infancy	9
c.4234C>T	p.L1412F	TM Domain 3	1	23	*CACNA1F*	L1079P	Night blindness	9
c.4255A>G	p.K1419E	TM Domain 3	1	24	*CACNA1C*	E1115K	BrS	9
c.4258G>C	p.G1420R	TM Domain 3	1	24	*SCN1A*	G1433R	Dravet syndrome	9
					*SCN1A*	G1433E	Myoclonic epilepsy of infancy	9
					*SCN1A*	G1433V	Dravet syndrome	9
c.4283C>T	p.A1428V	TM Domain 3	1	24	*SCN1A*	A1441P	Myoclonic epilepsy of infancy	9
c.4321G>C	p.E1441Q	TM Domain 3	1	25	*CACNA1A*	G1483R	Episodic ataxia	9
					*SCN1A*	E1454K	Dravet syndrome	9
c.4342A>C	p.I1448L	TM Domain 3	1	25	*SCN1A*	L1461I	Myoclonic epilepsy of infancy	9
c.4343T>C	p.I1448T	TM Domain 3	1	25	*SCN1A*	L1461I	Myoclonic epilepsy of infancy	9
c.4346A>G	p.Y1449C	TM Domain 3	1	25	*SCN1A*	Y1462C	Myoclonic epilepsy of infancy	9
					*CACNA1A*	F1491S	Episodic ataxia	9
					*SCN1A*	Y1462H	Dravet syndrome	9
c.4387A>T	p.N1463Y	TM Domain 3	1	25	*SCN1A*	N1476K	Dravet syndrome	9
c.4402G>T	p.V1468F	TM Domain 3	1	25	*SCN4A*	V1293I	Paramyotoniacongenita	9
c.4573G>A	p.V1525M	TM Domain 4	1	27	*SCN1A*	V1538I	Dravet syndrome	9
c.4642G>A	p.E1548K	TM Domain 4	3	27	*SCN1A*	E1561K	Dravet syndrome	9
c.4747C>T	p.R1583C	TM Domain 4	2	27	*SCN1A*	R1596C	Cryptogenic focal epilepsy	9
					*SCN1A*	R1596L	Dravet syndrome	9
					*SCN1A*	R1596H	Generalised epilepsy with febrile seizures	9
c.4748G>A	p.R1583H	TM Domain 4	1	27	*SCN1A*	R1596C	Cryptogenic focal epilepsy	9
					*SCN1A*	R1596L	Dravet syndrome	9
					*SCN1A*	R1596H	Generalised epilepsy with febrile seizures	9
c.4981G>C	p.G1661R	TM Domain 4	1	28	*SCN1A*	G1674R	Myoclonic epilepsy of infancy	9
c.4981G>A	p.G1661R	TM Domain 4	2	28	*SCN1A*	G1674R	Myoclonic epilepsy of infancy	9
c.5015C>A	p.S1672Y	TM Domain 4	2	28	*SCN1A*	A1685D	Myoclonic epilepsy of infancy	9
					*SCN1A*	A1685V	Febrile seizures	9
c.5134G>A	p.G1712S	TM Domain 4	1	28	*SCN1A*	G1725C	Dravet syndrome	9

Forty-five out of 122 novel missense variants identified in 2111 unrelated BrS patients^5^ were annotated. In addition, 26 of the 70 *SCN5A* missense variants previously reported to be pathogenic were annotated (see supplementary table S2, available online only).

*SCN5A* coordinates given with respect to transcripts NM_198056/NP_932173 (Refseq), ENST00000333535/ENSP00000328968 (Ensembl), LRG_289t1/LRG_289p1 (Locus Reference Genomic).

BrS, Brugada syndrome; CDS, coding DNA sequence; TM, transmembrane.

Three variants that are detected in healthy controls in these two studies are predicted to be pathogenic using our approach, and are considered false positives for the purpose of this study. *RYR2*-M2389 L was identified in one of 200 controls. This variant is found in the central domain hotspot of *RYR2* and is not identified in any of the 1000 Genomes phase 1 samples. *SCN5A*-D596G was identified in one of 1300 controls. Located in interlinker domain I–II, this variant is also absent in the phase 1 1000 Genomes data. *SCN5A*-S216 L has conflicting reports as to its pathogenicity—it has been linked to LQTS, atrial fibrillation and dilated cardiomyopathy,^21–23^ but has also been identified in healthy controls^8^ and in low frequencies in dbSNP populations. Its exact role in causing disease is therefore still unclear. Although we consider these variants as false positives here, in the absence of functional data to the contrary, at least some may be functionally deleterious in controls with non-penetrant disease or incomplete phenotype data. Importantly, controls did not undergo any provocation testing (eg, ajmaline challenge for BrS, and exercise test or adrenaline challenge for CPVT), which is used to make a diagnosis in some forms of these diseases and hence latent disease was not excluded.

Paralogue annotation has previously been validated in eight LQTS proteins.^10^ Sites predicted to be disease associated by paralogue annotation were highly enriched for known disease-causing variants, with very few false positives. Applying the approach to *RYR2* and *SCN5A* variants again demonstrates enrichment for known disease-associated residues (table 4). Two hundred and seventy-five *RYR2* residues were annotated with paralogue mutations: if distributed at random, eight would be expected to coincide with known pathogenic residues. We observe 35 known disease-associated residues: a 4.4-fold enrichment. Similarly, 113 disease-associated *SCN5A* residues are annotated by paralogue mutations, a 1.4-fold increase over the 81 annotations expected by random distribution. There is also a significant depletion of known benign residues in *SCN5A* (expected, six; observed, one). The positive predictive value (PPV) of paralogue annotation assessed in the current study is 98.7%, in keeping with previous findings in LQTS genes (98.4% PPV).^10^ By comparison, of 519 known missense variants in *RYR2* and *SCN5A* predicted by SIFT to be deleterious, 500 are correct (variants with unambiguous evidence of pathogenicity) with 19 false positives (benign variants), yielding a PPV of 96.5%. Similarly, of 518 variants predicted to be damaging by Polyphen, 497 are correct with 21 false positives, with a PPV of 95.9%.

**Table 4:** Paralogue annotation accurately identifies disease-associated residues in *RYR2* and *SCN5A*

Protein		Pathogenic	Benign	Uncertain	Unannotated	Total
*SCN5A*	Published observations	368	28	60	1560	2016
	Paralogue annotations: observed (expected)	113 (81)	1 (6)	6 (13)	321 (341)	441
*RYR2*	Published observations	139	20	71	4737	4967
	Paralogue annotations: observed (expected)	35 (8)	1 (1)	1 (4)	238 (262)	275

Distribution of paralogue annotations across the amino acid residues of the *SCN5A* and *RYR2* proteins. ‘Published observations’ shows the number of amino acid residues with known missense variants, classified as pathogenic, benign or uncertain, and the number of residues at which missense variation has not previously been observed (unannotated). ‘Uncertain’ refers to dbSNP variants without clinical information or residues with variants with conflicting reports as to pathogenicity. ‘Paralogue annotations observed’ shows the number of residues of each class that are annotated by variants in paralogues, and which would therefore be expected to be sites of pathogenic variation. ‘Paralogue annotations expected’ shows the number of residues in each class that would be expected to be annotated if paralogue annotation was random, with no predictive value. Variants annotated in this way are highly enriched for pathogenic variation in both genes (2×2 Fisher's exact test p=0.0009), with a positive predictive value (PPV) of 98.7%. 559 previously unannotated residues (321 in *SCN5A*, 238 in *RYR2*) are identified as putative disease-associated residues.

Importantly, the paralogue approach is informative for a large number of residues at which no variation has previously been reported, when variation detected in clinical testing would otherwise be reported as a ‘variant of unknown significance’. Paralogue annotation is informative for 238 unannotated *RYR2* residues and 321 unannotated *SCN5A* residues, and was informative for more than one-third of novel variants identified in these large clinical cohorts. It is expected that any missense variants identified in these residues in CPVT or BrS patients are likely to be disease causing.

### Distribution of variation across protein domains

**Figure 3:**
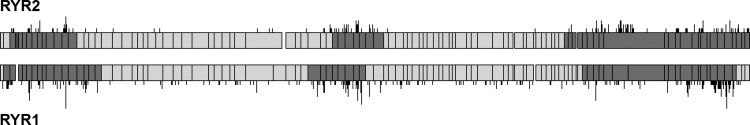
Disease-causing variation in human ryanodine receptors, *RYR1* and *RYR2*. An alignment of *RYR1* and *RYR2* reveals the structural similarity of the proteins and homologous clustering of pathogenic variation in these related proteins. The protein is represented in light grey, with reported ‘mutation hotspots’ marked in dark grey, and exon boundaries highlighted. The locations of missense variants previously reported to be pathogenic are shown with black lines above and below the protein graphic—longer lines indicate more than one pathogenic DNA variant affecting the same protein residue.

Protein regions that are known to be enriched for pathogenic variation were also enriched in paralogue annotations (compared with expectation under a random distribution; table 5). One hundred and seventy-two of the 275 paralogue mutations mapped to *RYR2* occur in the three mutations hotspots, an enrichment factor of 1.52. Similarly, 227 of the 441 paralogue mutations mapped to *SCN5A* occur in the four transmembrane domains, an enrichment factor of 1.57. As might be expected, disease-causing variants display similar domain distributions across the protein families: *RYR1* has similar overlapping mutation hotspots to *RYR2* (figure 3) and 75.8% of the missense mutations in the voltage-gated sodium and calcium channel paralogues of *SCN5A* occur in their transmembrane domains, despite these regions only accounting for 50.5% of the protein length.

**Table 5:** Annotation across protein domains of *RYR2* and *SCN5A*

		Known missense variants			Paralogue mappings	
Gene	Protein domains	Pathogenic	Benign	Uncertain	Actual	Enrichment
*SCN5A*	N-terminus	27	2	6	14	0.51
	Transmembrane	277	8	26	355	1.57
	Interlinker domains	99	25	35	41	0.31
	C-terminus	40	5	16	31	0.58
	Total	443	40	83	441	
*RYR2*	Hotspots	134	10	17	157	1.52
	Outside hotspots	20	10	59	118	0.69
	Total	154	20	76	275	

Distribution of known variants and paralogue mutation annotations across protein domains of *SCN5A* and mutation hotspots of *RYR2*. There is significant enrichment of both known pathogenic mutations and paralogue mutation mappings in the protein regions recognised to be susceptible to pathogenic variation, that is, the trans-membrane domains of *SCN5A* and the three mutation hotspots of *RYR2*.

### Reciprocal paralogue annotation: *RYR1*

This technique is also applicable in a reciprocal fashion, that is, arrhythmia-causing mutations in *RYR2* and *SCN5A* can be used to interpret novel variants found in any of the paralogues analysed in this study. As an example, we have mapped all the mutations in *RYR2* that cause CPVT and other cardiac diseases onto *RYR1*, which can be used to assess novel missense variants found in patients with malignant hyperthermia or central core disease.

As expected, residues predicted to be intolerant of variation by paralogue annotation are indeed highly enriched for known disease-associated variants in *RYR1* (4.4-fold enrichment; observed, 35; expected, eight). None of the four *RYR1* residues that host missense variants with a MAF greater than 0.01 in 1000 Genomes (that are therefore presumably benign) were annotated, giving a notional PPV of 100%. In addition, 99 previously unannotated *RYR1* residues are predicted to be intolerant of variation, of which 22 lie outside the traditional *RYR1* mutation hotspots.

### Web-based paralogue annotation application

**Figure 4:**
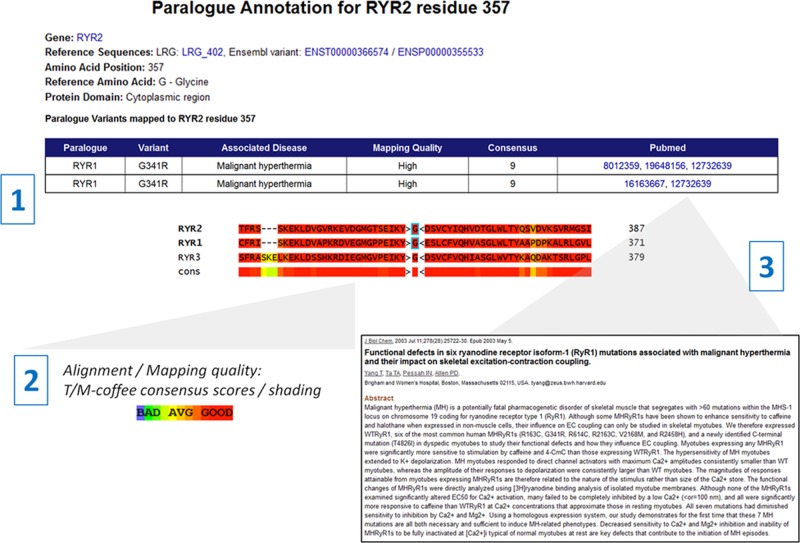
A web-based application makes paralogue annotation easily accessible for genes causing inherited arrhythmia syndromes. A web-based application is available at http://cardiodb.org/Paralogue_Annotation/. Users enter the position of a novel variant using complementary DNA or protein coordinates: in this example a substitution has been found in *RYR2*, affecting glycine residue 357.^1^ This residue maps to *RYR1* residue 341, and two cDNA variants at that location (c.1021G>A and c.1021G>C) that each cause substitution of Arg for Gly at that position have been reported to cause malignant hyperthermia. Users should check the alignment quality—here the mapping quality is high: the surrounding region is highly homologous, the reference amino acid is the same in both proteins, and the alignment has a high consensus score.^2^ Pubmed links give access to the reports relating to the paralogue mutation(s), 3 allowing users to assess the quality of evidence for pathogenicity. Here functional characterisation has been performed on this variant in the highlighted publication, adding confidence that the variant is disease causing and the residue is intolerant of variation in both *RYR1* and its paralogue *RYR2*.

We have developed a web-based tool to allow researchers and clinicians to identify paralogue annotations for novel variants to inform pathogenicity. This is available for *RYR2* and *SCN5A*, as well as other LQTS-associated genes previously reported. Users input the coordinates of a novel variant (protein or complementary DNA coordinates) in a gene of interest, and any paralogue mutations associated with that residue are returned, together with fully referenced details of any known (pathogenic or benign) missense variants in the query gene. The quality of the mapping is crucial when using paralogue annotation to predict pathogenicity. The web interface will assign each mapping a high, medium or low quality rating (defined using the alignment consensus, reference amino acids in the query and paralogue proteins and number of mappings as described in the Methods section above). In addition, the segment of the multiple sequence alignment around the mapping is displayed to allow users to assess the degree of homology visually between the proteins at that residue, coloured by the consensus between the different alignment algorithms used by the M-Coffee and T-Coffee packages. Finally, links to the publications describing the pathogenic paralogue mutations are available, allowing users to assess how well the mutation has been characterised in the paralogue-associated disease. The application is shown in figure 4.

## Discussion

In this study we have shown that over one-third (36%) of novel missense variants identified in large cohorts of patients with CPVT (*RYR2*) and BrS (*SCN5A*) can be annotated by paralogue mutations. This additional evidence of pathogenicity may allow us to reclassify as disease causing up to one-third of variants currently reported as ‘variants of unknown significance’, with a positive PPV exceeding 98%.^10^ There is a bias towards reporting pathogenic variants over benign variants in the literature, and although we have supplemented literature reports with data from 1000 Genomes and the Exome Sequencing Project, it is possible that these PPV values may be overestimated. However, as these data derive from clinically observed genotype–phenotype relationships within closely related protein families, rather than theoretical predictions based on the sequence and structure of much more distantly related proteins, we have confidence in this approach and these annotations.

In a clinical setting, the identification of a novel single amino acid substitution in a gene associated with inherited disease is generally insufficient to report the variant as disease causing. This is due to the relatively high prevalence of rare, benign variants in healthy controls in many genes. Missense variants have been shown to be present in 6% of controls in *RYR2*^4^ and in 2.7% of controls in *SCN5A.*^8^ Segregation studies and functional biochemical characterisation are powerful, but resource intensive and are not always applicable. Computational predictions are attractive and widely applicable, but while informative they are not sufficiently accurate for robust clinical reporting. It therefore behoves us to make full use of available reports linking human variation to clinical phenotype.

When a variant in a disease gene has previously been observed, either in association with disease or in apparently healthy individuals, this provides invaluable additional information for clinical reporting. However, there is a huge amount of additional data linking genetic variation to clinical phenotype in paralogues of disease genes that is not presently used. By mapping functionally equivalent amino acid residues across protein families, and transferring genotype–phenotype annotations between related proteins, we can leverage this wealth of existing data to aid our interpretation of the significance of novel variants in a clinical setting.

The accuracy of this method depends on the quality of the protein sequence alignment (to ensure that aligned amino acids can be confidently regarded as functionally equivalent) and the quality of the evidence relating genotype to phenotype for the paralogue variant. For the alignment, appropriate paralogues should be carefully and manually chosen, while we have described methods above to ensure that only mappings of sufficient quality are noted. In addition, our web application allows users to assess the quality of the alignment visually at the mapping residue. The web application also provides Pubmed links to allow researchers to assess the quality of the reports linking the paralogue variants to disease—a functionally characterised variant, or one that has been shown to segregate with disease, is more informative than a variant simply observed once in an affected individual. It is critical for researchers to consider this evidence carefully to avoid inferring pathogenicity from erroneous reports and also check that the direction of effect of the variant in the paralogue is compatible with the observed phenotype in the gene of interest.

The sensitivity of this approach depends on the availability of known, pathogenic mutations in the paralogues of genes of interest. The lack of paralogue annotation for a novel variant does not imply that the variant is non-pathogenic, simply that a disease-causing mutation in the equivalent paralogue residue has not yet been observed. As genetic testing becomes more widely available across the whole spectrum of inherited disease, for example, through the ongoing development of sequencing technologies, we expect the number of informative paralogue mutations to expand greatly, increasing the sensitivity of this method. Here, an additional 238 residues in *RYR2* (a 171% increase compared with previous reports), and 321 residues in *SCN5A* (87% increase) are putatively annotated as intolerant of variation.

Paralogue annotation is designed to interpret individual variants in monogenic disease and does not currently help to clarify the genetics of complex multi-alleleic disease. There have been recent reports linking common genetic variation with predisposition to BrS,^24^ and there is a debate about the role of genetic testing in this condition. However as BrS does segregate as a monogenic Mendelian trait in some pedigrees and most clinicians still feel that genetic testing of *SCN5A* is of value, paralogue annotation can help to interpret these novel variants.

Some regions of *RYR2* and *SCN5A* are enriched for disease-associated variants, yet contain relatively few benign polymorphisms, so that variants in these regions have a higher-than-average previous probability of pathogenicity. While this is to some extent informative, the accuracy and sensitivity of a purely domain-based prediction are limited, as many pathogenic and benign variants do not follow this trend. Paralogue annotation provides a higher resolution annotation, down to the level of the individual amino acid. It is also not limited to any specific domains or protein regions and is less likely to mis-annotate a rare benign variant as pathogenic given the high PPV scores we have seen in this and previous studies.

In *RYR2*, 134 of the 154 known pathogenic missense mutations occur within the recognised mutation hotspots (figure 3). However, 10 known benign missense variants also occur in these regions, suggesting that novel variants found here cannot be definitively classified as disease causing. In our study we found that 57% of the paralogue annotations of *RYR2* occur in the mutation hotspots. This is due to the fact that *RYR1* contains equivalent, and largely overlapping, mutation hotspots (figure 3). Pathogenic mutations in *RYR1* are, however, less concentrated in these hotspots than in *RYR2*, which allows us to annotate an additional 114 residues outside conventional *RYR2* hotspots. It is likely that this clustering of mutations is at least partly caused by sequencing bias, that is, often only exons with known mutations are sequenced in genetic testing. The increasing use of next generation sequencing technologies allows laboratories to sequence all the exons of ryanodine receptors and is likely to expand the numbers of mutations found outside the hotspots of both *RYR1* and *RYR2* for their respective diseases—paralogue annotation will be able to utilise all of these findings to assess variants in either gene.

Pathogenic mutations are not as discreetly clustered in *SCN5A* as in *RYR2*. However, variation in the transmembrane regions is rare in apparently healthy individuals, and substitutions in these regions have been reported to have an 88% probability of pathogenicity when found in clinically affected individuals.^8^ Here, over 80% of disease-causing variants in *SCN5A* paralogues map to transmembrane residues, providing further evidence that these domains are functionally important and intolerant of variation. Most importantly, paralogue annotation also annotates some variants at residues outside these regions, whose significance would otherwise be unappreciated.

Paralogue annotation of variants is widely applicable: approximately half of all disease-associated genes have one or more paralogues with disease-causing variants.^10^ The technique can also be applied in a reciprocal manner, as illustrated here with *RYR1* and *RYR2*, and our web-based application will be informative for researchers and clinicians interested in a range of diseases mediated by ion channels and membrane excitability, such as malignant hyperthermia and central core disease, and as well as the cardiovascular genetics community.

## Conclusion

This study demonstrates an accurate and widely applicable approach to interpret novel missense variants, here applied to annotate variants in CPVT (*RYR2*) and BrS (*SCN5A*). This is informative for more than one-third of novel variants in these genes, and may provide sufficient evidence to report these variants as disease causing, rather than as ‘variants of unknown significance’, with a positive PPV of 98.7%. This approach is based on clinical genotype–phenotype relationships in humans, rather than computational prediction, giving further confidence in its application. While this method will not be applied in isolation, due to our incomplete inventory of pathogenic variation in paralogues, it provides an invaluable additional tool for clinicians and researchers. Our web-based application provides a user-friendly implementation of this technique for immediate application in the interpretation of novel variants, with an initial focus on inherited arrhythmia syndrome genes, together with an annotated compendium of previously reported missense variants in these genes.

## Supplementary Material

Web tables
