# Supplementary material for: Paralogue annotation identifies novel pathogenic variants in patients with Brugada syndrome and catecholaminergic polymorphic ventricular tachycardia
Source: J Med Genet. 2013 Oct 17;51(1):35–44. doi: 10.1136/jmedgenet-2013-101917 (PMC3888601; doi:10.1136/jmedgenet-2013-101917)
Supplement: Web tables [file jmedgenet-2013-101917-s1.pdf]

Supplementary Table 1: Paralogue annotation for missense variants, identified in CPVT cohort, that have been previously or subsequently been reported to be associated with CPVT.

RYR2 variant CDS	RYR2 variant Protein	Region	Cases (n=155)	Exon	Paralogue	Paralogue mutation	Paralogue disease	Consensus
c.7159G>A	p.A2387T	Central hotspot	3	47	RYR1	A2421P	Congenital myopathy with cores	9
c.7202G>A	p.R2401H	Central hotspot	1	47	RYR1	R2435H	Central core disease	9
					RYR1	R2435L	Central core disease	9
c.7207G>A	p.A2403T	Central hotspot	1	47	RYR1	A2437V	Malignant hyperthermia	9
c.14011G>C	p.G4671R	Channel hotspot	1	97	RYR1	G4743D	Multiminicore disease	9
c.14311G>A	p.V4771I	Channel hotspot	2	100	RYR1	V4842M	Congenital myopathy with cores	9

Supplementary Table 2: Paralogue annotations for missense variants, identified in BrS patients, that have previously/subsequently been reported as pathogenic.

SCN5A variant CDS	Protein	Region	Cases (n=2111)	Exon	Paralogue	Paralogue variant	Paralogue disease	Consensus
c.311G>A	p.R104Q	N-terminus	3	3	SCN1A	R101W	Myoclonic epilepsy of infancy	7
					SCN1A	R101Q	Myoclonic epilepsy of infancy	7
c.310C>T	p.R104W	N-terminus	2	3	SCN1A	R101W	Myoclonic epilepsy of infancy	7
					SCN1A	R101Q	Myoclonic epilepsy of infancy	7
c.361C>T	p.R121W	N-terminus	1	3	SCN1A	R118S	Myoclonic epilepsy of infancy	9
c.665G>A	p.R222Q	TM domain 1	1	6	SCN4A	R222W	Hypokalaemic periodic paralysis	9
c.673C>T	p.R225W	TM domain 1	3	6	SCN2A	R223Q	Neonatal-infantile seizures	9
					SCN4A	R225W	Non-dystrophic myotonia	9
c.677C>T	p.A226V	TM domain 1	2	6	SCN1A	A223E	Dravet syndrome	9
c.1066G>A	p.D356N	TM domain 1	8	9	SCN1A	D366E	Myoclonic epilepsy of infancy	9
c.1099C>T	p.R367C	TM domain 1	2	9	SCN1A	R377Q	Generalized epilepsy with febrile seizures	9
					SCN1A	R377L	Dravet syndrome	9
c.1100G>A	p.R367H	TM domain 1	6	9	SCN1A	R377Q	Generalized epilepsy with febrile seizures	9
					SCN1A	R377L	Dravet syndrome	9
c.1106T>A	p.M369K	TM domain 1	1	9	SCN1A	M379R	Dravet syndrome	9
c.2314G>A	p.D772N	TM Domain 2	1	15	CACNA1H	G848S	Childhood absence epilepsy	9
c.2423G>C	p.R808P	TM Domain 2	1	15	SCN1A	R859C	Generalized epilepsy with febrile seizures	9
					SCN4A	R669H	Hypokalaemic periodic paralysis	9
					CACNA1S	R528G	Hypokalaemic periodic paralysis	9
					CACNA1S	R528H	Hypokalaemic periodic paralysis	9
					CACNA1A	R583Q	Hemiplegic migraine and ataxia	9

					SCN1A	R859H	Generalized epilepsy with febrile seizures plus	9
c.2516T>C	p.L839P	TM Domain 2	1	16	SCN1A	L890P	Dravet syndrome C ?	9
c.2632C>T	p.R878C	TM Domain 2	1	16	SCN1A	R931C	Myoclonic epilepsy of infancy	8
					SCN9A	R896Q	Congenital indifference to pain	8
					SCN1A	R931H	Epilepsy ?	8
c.3673G>A	p.E1225K	TM Domain 3	4	21	SCN1A	E1238D	Myoclonic epilepsy of infancy	9
c.3694C>T	p.R1232W	TM Domain 3	3	21	SCN1A	R1245Q	Myoclonic epilepsy of infancy	7
c.3784G>A	p.G1262S	TM Domain 3	1	21	SCN1A	G1275V	Dravet syndrome	9
c.3823G>A	p.D1275N	TM Domain 3	3	21	CACNA1F	D944Y	Night blindness, congenital stationary, incomplete	9
					SCN1A	D1288N	Dravet syndrome C ?	9
c.3995C>T	p.P1332L	TM Domain 3	1	23	SCN4A	P1158S	Hypokalaemic periodic paralysis	9
					SCN9A	P1308L	Erythermalgia, primary	9
c.4018G>A	p.V1340I	TM Domain 3	1	23	SCN1A	V1353L	Generalized epilepsy with febrile seizures plus	9
c.4145G>T	p.S1382I	TM Domain 3	1	23	SCN1A	T1394I	Dravet syndrome C ?	4
c.4313C>T	p.P1438L	TM Domain 3	1	25	SCN1A	P1451L	Myoclonic epilepsy of infancy	9
					SCN1A	P1451S	Dravet syndrome C ?	9
c.4501C>G	p.L1501V	Interdomain Linker III-IV	1	26	SCN1A	L1514S	Dravet syndrome	2
c.4868G>A	p.R1623Q	TM Domain 4	1	28	SCN1A	R1636Q	Lennox-Gastaut syndrome	6
					SCN4A	R1448S	Paramyotonia congenita	6
					SCN4A	R1448C	Paramyotonia congenita	6
					SCN4A	R1448H	Paramyotonia congenita	6
					SCN4A	R1448P	Myotonia	6
					SCN4A	R1448L	Paramyotonia congenita	6
					CACNA1F	R1296S	Night blindness, congenital stationary, incomplete	6

					CACNA1A	R1662H	Episodic ataxia 2	6
c.4978A>G	p.I1660V	TM Domain 4	5	28	CACNA1A	V1696I	Hemiplegic migraine	9
					CACNA1A	V1696F	Hemipl. migraine/alternating hemipl. of childhood	9
					SCN1A	I1673T	Dravet syndrome C ?	9
c.5218G>A	p.G1740R	TM Domain 4	1	28	SCN1A	G1754R	Dravet syndrome	3
